# Biodegradation of Mycotoxins: Tales from Known and Unexplored Worlds

**DOI:** 10.3389/fmicb.2016.00561

**Published:** 2016-04-25

**Authors:** Ilse Vanhoutte, Kris Audenaert, Leen De Gelder

**Affiliations:** Department of Applied BioSciences, Faculty Bioscience Engineering, Ghent UniversityGhent, Belgium

**Keywords:** detoxification, microorganisms, mycotoxins, biodegradation, metabolite

## Abstract

Exposure to mycotoxins, secondary metabolites produced by fungi, may infer serious risks for animal and human health and lead to economic losses. Several approaches to reduce these mycotoxins have been investigated such as chemical removal, physical binding, or microbial degradation. This review focuses on the microbial degradation or transformation of mycotoxins, with specific attention to the actual detoxification mechanisms of the mother compound. Furthermore, based on the similarities in chemical structure between groups of mycotoxins and environmentally recalcitrant compounds, known biodegradation pathways and degrading organisms which hold promise for the degradation of mycotoxins are presented.

## Introduction

The presence of mycotoxins is inherent to many food and feed products worldwide (Bhat et al., 2010; Marroquín-Cardona et al., 2014). Hallmarks of their presence and their impact on animal and human health are encountered throughout history. Ergotism, also known as “St. Anthony's fire” occurred in several areas in Europe during the tenth century (Schiff, 2006) and was caused by the consumption of rye containing ergot alkaloids, produced by the fungus *Claviceps purpurea* (Bové, 1970; Beardall and Miller, 1994). In Siberia, a delayed harvest due to the second world war resulted in grains heavily contaminated with trichothecenes produced by *Fusarium* spp. People later consuming the grain were afflicted with number of nonspecific disorders and mortality mounted up to 10% (Manahan, 2002). In 1962, 100,000 turkeys died in London of Turkey X disease, linked to aflatoxins from *Aspergillus flavus* (Binder, 2007). These examples mentioned above illustrate the acute impact of high loads of singular mycotoxins on human and animal health. However, longtime exposure to low concentrations of mycotoxins also entail chronic toxicities which often result in non-specific symptoms, difficult to track-and-trace down to mycotoxins. These toxicities include estrogenic gastrointestinal, urogenital, vascular, kidney, and nervous disorders. Some mycotoxins are carcinogenic or immuno-compromising, and as such also promote the development of infectious diseases (Peraica et al., 1999; Hussein and Brasel, 2001; Creppy, 2002; Richard, 2007; Da Rocha et al., 2014).

For many years the research community focused on the occurrence of singular mycotoxins but nowadays scientific interest shifts to studies involving multiple mycotoxins. This new approach is highly relevant as large scale multi-toxin surveys show that a number of mycotoxins tend to co-occur with other sometimes structurally not-related mycotoxins (Gerding et al., 2014; Storm et al., 2014; Vanheule et al., 2014; and many more). In addition, mycotoxins are known to have additive and synergistic effects on human- and animal health (Alassane-Kpembi et al., 2013; Klaric et al., 2013; Clarke et al., 2014).

Research efforts progressively increase to develop mitigation strategies based on risk monitoring, risk characterization, prevention, intervention, and remediation strategies for multiple mycotoxins, which start from critical points along the production chain comprising field, storage, processing, and transportation. However, monitoring and good agricultural, storage, and transportation practices along with an effective Hazard Analysis and Critical Control Point approach do not completely prevent mycotoxin presence in the food or feed chain (Bhat et al., 2010). Decontamination technologies then offer a last resort to salvage contaminated batches along the production chain.

Decontamination strategies to reduce mycotoxins in food- and feed commodities are technologically diverse and based on physical, chemical, or biochemical principles. Some physical processes aim to remove highly contaminated fractions from bulk material (Bullerman and Bianchini, 2007; Cheli et al., 2013; Kaushik, 2015) through sorting (Scudamore et al., 2007), milling (Castells et al., 2007; Khatibi et al., 2014), dehulling (Fandohan et al., 2006; Rios et al., 2009; Matumba et al., 2015), cleaning (van der Westhuizen et al., 2011), heating, irradiation, or combinational approaches (Fandohan et al., 2005; Matumba et al., 2015). Another physical removal strategy is the use of inorganic or organic mycotoxin binders (Ramos et al., 1996; Kolosova and Stroka, 2011). Although these adsorbing binders have some promising features, some may have adverse nutritional effects due to binding of vitamins and minerals (Huwig et al., 2001; Yiannikouris et al., 2006) or reducing the efficacy pharmacokinetics of antibiotics (De Mil et al., 2015).

Chemical remediation strategies involve the conversion of mycotoxins via chemical reactions. Ammoniation (Norred et al., 1991), alkaline hydrolysis, peroxidation, ozonation, and the use of bisulphites are reported to be effective on one or more mycotoxins but a detailed insight into the toxicity of eventual end products or the impact on palatability and nutritive quality is questionable.

Microbial based methods comprise mycotoxin decomposition, transformation, or adsorption. The latter strategy has already been mentioned under physical measures and will not be considered in detail in this review. Focus in this review will be on transformation and biodegradation of the main mycotoxins by microorganisms. Although there are some excellent reviews on biodegradation (Zinedine et al., 2007; Wu et al., 2009; Awad et al., 2010; Jard et al., 2011; Devreese et al., 2013; McCormick, 2013; Hathout and Aly, 2014; Adebo et al., 2015), this review is timely because of two reasons:

Firstly, studies often wrongly identify biodegradation with detoxification, or do not test for toxicity of potential metabolites. Indeed, not all transformation or degradation products are detoxification products. This is nicely illustrated for aflatoxins and zearalenone (ZEN). Aflatoxin M1 (AFM1) is the hydroxylated metabolite of AFB1 and is categorized as *possible carcinogenic to humans* (Group 2B) by the International Agency for Research on Cancer (IARC; IARC, 2002). Aflatoxicol (or aflatoxin R_0_), a reduction product of AFB1, has been detected as degradation product by *Corynebacterium rubrum, Aspergillus niger, Trichoderma viride, Mucor ambiguous*, and *Dactylium dendroides* (Mann and Rehm, 1976; Wong and Hsieh, 1976). However, Karabulut et al. (2014) concluded that AFB1 and aflatoxicol have similar potency to form an exo-epoxide analog which can bind to DNA. Assessing the ZEN biodegradation capacity of several microorganisms, Hahn et al. (2015) found that many strains were able to convert ZEN to α- and/or β-ZEL, showing similar estrogenic activity compared to ZEN. Aerobic and anaerobic degradation to other uncharacterized metabolites with unidentified toxicity was obtained as well. These results demonstrate the importance of *in vitro* experiments to critically screen agents claiming mycotoxin detoxification.

Secondly, the available set of mycotoxin degrading microorganisms is limited and their performance is often doubtful when considering multiple mycotoxin degradation. This issue was also nicely illustrated by Hahn et al. (2015). Using an *in vitro* screening approach, 20 commercially available agents claiming mycotoxin detoxification were tested for their efficacy to inactivate and/or degrade the two structurally not related mycotoxins DON or ZEN. The majority of the agents were not effective or converted the toxins to equally toxic metabolites. Only one of the products efficiently inactivated or degraded the two considered mycotoxins under the tested conditions.

New insights on actual microbial detoxification routes are needed and can be based on known biodegradation metabolisms of non-mycotoxins found in diverse microbial communities, which we chose to identify as “unexplored worlds” to be discovered for the mycotoxin research field. Indeed, many hazardous, undesirable, deleterious, or recalcitrant molecules in other research fields share structural analogies with diverse mycotoxins and are reported to be successfully degraded by microorganisms. These unexplored worlds may serve as resource for cutting edge research in the field of mycotoxin remediation or in the field of metagenomics screening surveys in search for new microbial degraders of mycotoxins.

In this review, We are not only focusing on *Fusarium* mycotoxins, but also on *Aspergillus, Penicillium*, and other mycotoxins. This is relevant as independently of the producing genus, mycotoxins often share key-chemical groups responsible for their toxicity and thus biodegrading organisms for one mycotoxin can have their relevance for other mycotoxins produced by distinct fungal genera.

## Toxicity and degradation of mycotoxins

In order to assess detoxification by microorganisms, it is important to pinpoint the actual groups within the chemical structure of each mycotoxin which infer the toxic effects (Table 1). Next to the main toxic structural groups occurring in mycotoxins, structural similarities between mycotoxins are also highlighted; aflatoxins and ochratoxins are both composed of a coumarin moiety, whereas the main structure of aflatoxins, ZEN and ochratoxins is based on a lactone ring (Table 1—Red). Carboxyl derivatives (ester bonds), often playing a role in toxicity, are frequently present, as well in the lactone, as in side groups (Table 1—Red) (observed in fumonisins, ZEN, ochratoxins, and acylated trichothecenes). Each mycotoxin is further characterized concerning specific groups responsible for its toxicity (Table 1—Blue).

**Table 1:** **Chemical structural groups inferring toxicity in mycotoxins**.

**Mycotoxin group**	**Side groups**	**Main toxic structural groups**	**References**
**AFLATOXINS**			
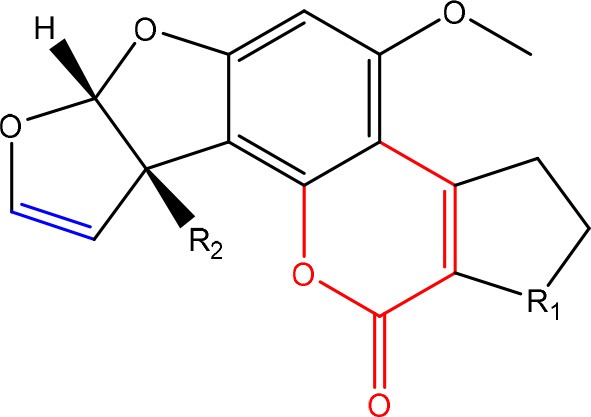	R_1_ = C = O,-(C = O)-O- or C-OH	Lactone ring	Lee et al., 1981
	R_2_ = H or OH	Double bond in difuran ring moiety	Wogan et al., 1971
**FUMONISINS**			
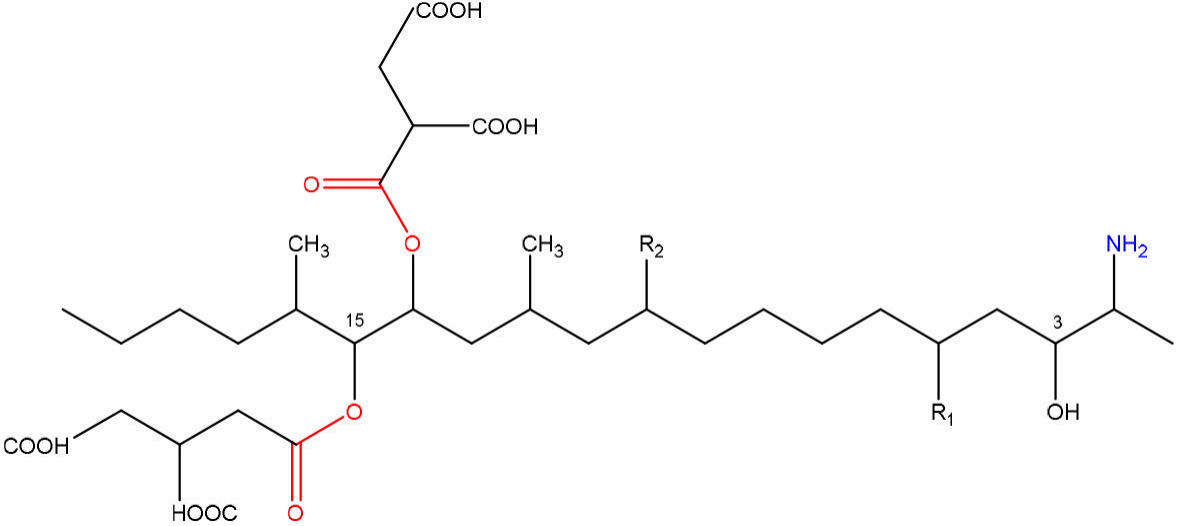	R_1_ = H or OH	Two tricarballylic acid side chains	Abbas et al., 1993b; Merrill et al., 1993b; Abbas et al., 1995; Voss et al., 1996b; Norred et al., 1997
	R_2_ = H or OH	Free amino group	Abbas et al., 1993b; Norred et al., 1997
**ZEARALENONES**			
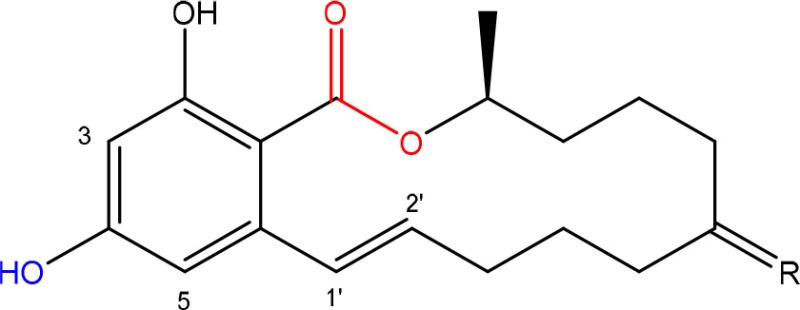	R = O or H, α-OH	Lactone ring	El-sharkawy S. and Abul-hajj Y. J., 1988
	1',2' = trans or dihydro	C-4 hydroxyl group	El-sharkawy S. H. and Abul-hajj Y. J., 1988
**TRICHOTHECENES**			
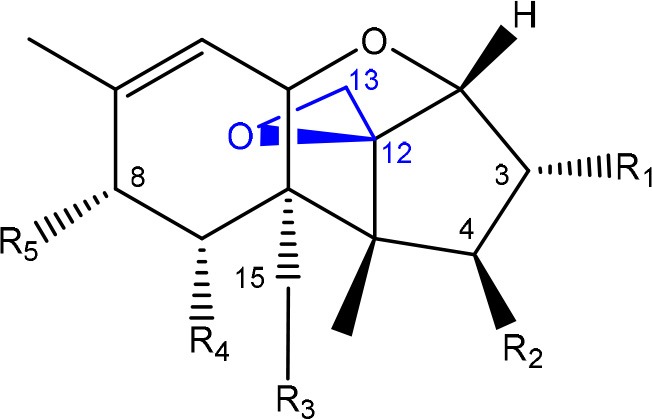	R_1_ = H or OH	Epoxide group	Zhou et al., 2008
	R_2_ = H, OH or OAc	Acylated side groups	Rocha et al., 2005
	R_3_ = OH or OAc		
	R_4_ = H or OH		
	R_5_ = H, OH, = O, -O-(C = O)-CH2-CH-(CH3)2 or -O-(C = O)-CH2-COH-(CH3)2		
**OCHRATOXINS**			
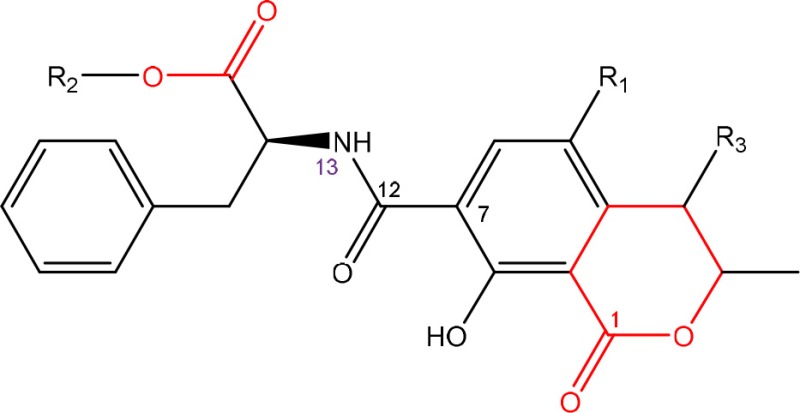	R_1_ = Cl or H	Isocoumarin moiety	Xiao et al., 1996
	R_2_ = H, methyl or ethyl	Carboxyl group of the phenylalanine moiety	
	R_3_ = H or OH	Cl group	

Red, carboxylic derivatives (lactone rings and ester bonds) in red; Blue, specific groups responsible for toxicity.

From our perspective, there are two ways in which detoxification of the mother compound in a degradation study can be confirmed: (i) the confirmation of reduced toxicity after degradation through one or more actual toxicity assays on particular organisms or cell lines, this is the most convincing proof; (ii) the detection and identification of detoxification products, for which in independent literature has been shown that they confer an lower toxicity to the mother compound. Of course, a combination of both ways provides the most holistic approach. The decreased toxicity of the degradation metabolites listed in Tables 2–**7** can therefore be found in Table 1 in Supplementary Material.

**Table 2:** **Degradation and/or detoxification products of fumonisins**.

**Degradation and/or detoxification product**	**Microorganism**	**References**
**REMOVAL OF TRICARBALLYLATE SIDE CHAINS AND AMINO GROUP**		
- By carboxylesterase and aminotransferase	*Sphingomonas* sp. ATCC 55552^*^	Duvick et al., 1998a; Heinl et al., 2011
		Patents: Duvick et al., 1998b, 2003
	*Sphingopyxis* sp. MTA 144	Täubel, 2005; Hartinger et al., 2009; Heinl et al., 2009, 2010
		Patent: Moll et al., 2014
- By carboxylesterase and oxidative deaminase	*Exophiala* sp^*^.	Duvick et al., 1998a; Blackwell et al., 1999
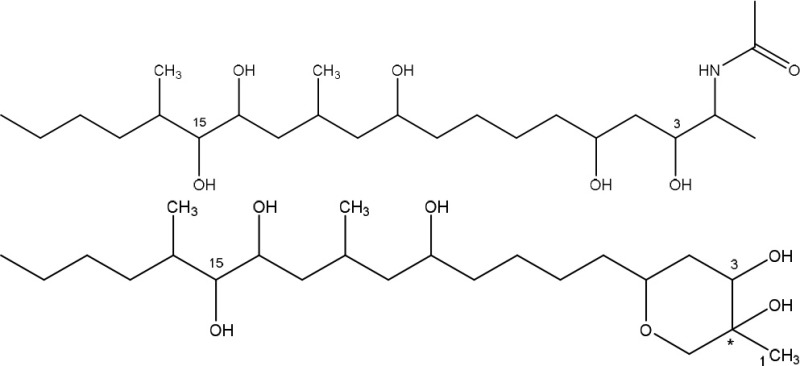		Patents: Duvick et al., 1998b, 2003
**ACCUMULATION OF (TENTATIVELY) HEPTADECANONE, ISONONADECENE, OCTADECENAL AND EICOSANE**		
	*Delftia/Comomonas* NCB 1492^#^	Benedetti et al., 2006
**REMOVAL OF AMINO GROUP**		
based on increased pH	*Bacillus* sp. and yeast strain	Camilo et al., 2000

* , growth on fumonisin as sole carbon source.

# , growth on fumonisin as sole carbon and nitrogen source.

### Fumonisins

#### Toxicity

Fumonisins (most importantly FB1, FB2), first described by Gelderblom et al. (1988), are mainly produced by *F. verticillioides* and *F. proliferatum* and are structurally similar to sphingolipid long-chain bases such as sphinganine and sphingosine. This feature is tightly related to their toxicity mechanism through the inhibition of the sphingolipid biosynthesis (Merrill et al., 1993a; Soriano et al., 2005) and exposure to fumonisins has been associated a wide variety of diseases in animals as reviewed by Voss et al. (2007), such as liver cancer in rats, equine leukoencephalomalacia, and porcine pulmonary edema.

Specifically, fumonisins are comprised of a 22 carbon aminopentol with two tricarballylate (TCA) side groups, where two structural groups are important in their toxicity mechanism (Table 1). Firstly, the unsubstituted primary amino group at C2 competitively inhibits ceramide synthase, thereby disrupting the *de novo* biosynthesis of ceramide and sphingolipid metabolism (Voss et al., 2007). This free primary amino group of fumonisin-like compounds is a prerequisite for ceramide synthase inhibition, since N-acetylation of FB1 diminished or removed the toxicity effects in rat liver slices (Norred et al., 1997) and in jimsonweed and several mammalian cell lines (Abbas et al., 1993b). Secondly, the TCA side groups seem to have varying effects on the toxicity. On the one hand, absence of these side groups has been found to reduce both phytotoxicity and mammalian cytotoxicity (Abbas et al., 1995), and the resulting corresponding aminopentol (AP1, AP2) backbones were only 30–40% (Norred et al., 1997) or 10% as potent as the parent toxins (Merrill et al., 1993b). In contrast, removal of the TCA side groups has also been shown to enhance cytotoxicity in certain mammalian cell lines (Abbas et al., 1993b) and AP1 displays renal toxicity comparable to that of FB1 (Voss et al., 1996a).

#### Degradation: organisms and pathways

Only a few microorganisms are known to degrade and thereby detoxify fumonisins (Table 2), mostly by removal of the TCA groups as well as the free amino group. Although none of these studies actually determined detoxification of fumonisin B1 by these microorganisms through *in vitro* assays, based on what is known regarding the role of the TCA groups and the free amino group in inferring the toxicity of FB1 (Table 1, Table 1 in Supplementary Material), we can safely assume detoxification was indeed achieved.

*Sphingomonas* sp. ATCC 55552 was isolated from field-grown, moldy maize kernels, and stalk tissue (Duvick et al., 1998a) and been shown to degrade fumonisin B1 through the consecutive action of a carboxylesterase (Duvick et al., 2003) and an aminotransferase (Heinl et al., 2011). The same pathway was found in *Sphingopyxis* sp. MTA 144 isolated from composted earth (Täubel, 2005), in which the gene cluster responsible for fumonisin degradation was identified with *fumD*, encoding the carboxylesterase and *fumI* encoding the aminotransferase (Hartinger et al., 2009; Heinl et al., 2009, 2010).

Degradation by *Exophiala* sp., also isolated from field-grown, moldy maize kernels, and stalk tissue (Duvick et al., 1998a), was shown to be conferred by a carboxylesterase and, in contrast to ATCC 55552 and MTA 144, by an oxidative deaminase. Two degradation products were identified: a new compound, 2-oxo-12,16-dimethyl-3,5,10,14,15-icosanepentol hemiketal, and in smaller amounts the N-acetylated aminopentol backbone (N-acetylAP1).

Strain NCB 1492, isolated from maize field soil and related to the *Delftia/Comamonas* group, gave rise to four tentative degradation products of fumonisin B1 (C_34_H_59_NO_15_): heptadecanone (C_17_H_34_O), isononadecene (C_19_H_38_), octadecenal (C_18_H_34_O), and eicosane (C_20_H_42_) (Benedetti et al., 2006). The first degradative steps are thought to occur extracellularly, with deamination (and possibly esterase) activities followed by a slower degradation of the aliphatic chain.

Insights into the detoxification of fumonisins can also be useful for mycotoxins produced by other fungal genera. In this light, we would like to draw the focus on the *Alternaria* toxins AAL-T_A_ en -T_B_, which share with fumonisins a distinct structural similarity and toxicity mechanism (Abbas et al., 1993a; Tsuge et al., 2013). Fumonisins have two TCA side chains esterified to the aminopentol backbone, whereas AALtoxins have only one TCA side group, and are therefore collectively referred to as sphinganine-analog mycotoxins. To the best of our knowledge, there have not been any reports of microbial strains capable of degrading AAL-toxins, but based on their structural similarity it is likely that fumonisin degrading organisms as described above might also be capable of degrading AAL-toxin.

### Zearalenone

#### Toxicity

ZEN is mainly produced by fungi belonging to the genus *Fusarium* such as *F. graminearum* and *F. culmorum* and possesses estrogenic activity in pigs, cattle and sheep (Zinedine et al., 2007). The toxicity of ZEN is mainly conferred by its lactone group and the free C-4 hydroxyl group (Table 1) which is necessary for binding the estrogen receptor (El-sharkawy S. H. and Abul-hajj Y. J., 1988). Many derivatives of ZEN are known and some exhibit a higher estrogenicity than the mother compound (Shier et al., 2001), such as α-zearalenol, α- and β-zearalanol, and zearalanone. Several studies described the microbial transformation of ZEN to such derivatives, but as they do not represent a true detoxification of the compound they are not discussed in this review. Also, cases in which no clear evidence is presented (yet) for true detoxification (e.g., *Pseudomonas* strains in Tan et al., 2014, 2015) are not discussed in detail.

#### Degradation: organisms and pathways

To date, two main detoxification mechanisms are known for ZEN, both cleaving a ring structure (Table 3). The lactone ring can be cleaved by several fungal species through two mechanisms. Degradation by *Gliocladium roseum* NRRL1859 (El-sharkawy S. and Abul-hajj Y. J., 1988) resulted in a 1:1 mixture of 1-(3,5-dihydroxyphenyl)-10′-hydroxy-1-undecen-6′-one, and 1-(3,5-dihydroxyphenyl)-6′-hydroxy-1-undecen-10′-one. Matthies et al. (2001) showed that production of the ZEN-degrading enzyme in *G. roseum* DSM 62726 was induced the highest by the derivatives zearalanol and α-zearalanol. Almost similarly, only the first metabolite was observed after degradation by a near isogenic strain of NRRL1859, *Clonostachys rosea* (synonym: *G. roseum*, teleomorph: *Bionectria ochroleuca*) IFO 7063 (Kakeya et al., 2002), resulting in the loss of estrogenic activity in MCF-7 cancer cells (Table 1 in Supplementary Materials), through the activity of a ZEN lactonohydrolase enzyme (zhd101) which catalyzes the hydrolysis of ZEN at the ester bond in the lactone ring, followed by spontaneous decarboxylation (Takahashi-Ando et al., 2004). Based on this knowledge, Popiel et al. (2014) searched a collection of *Trichoderma* and *Clonostachys* isolates for functional lactonohydrolase homologs, to find a functional ZEN lactonohydrolase in mycoparasitic *Trichoderma aggressivum*. A similar pathway might also exist in *Bacillus* sp., as cell culture extracts of *B. natto* CICC 24640 and *B. subtilis* 168 showed complete degradation of ZEN in conjunction with CO_2_-emmission, indicative of decarboxylation (Tinyiro et al., 2011).

**Table 3:** **Degradation and/or detoxification products of ZEN**.

**Degradation and/or detoxification product**	**Microorganism**	**References**
**CLEAVAGE OF THE LACTONE RING**		
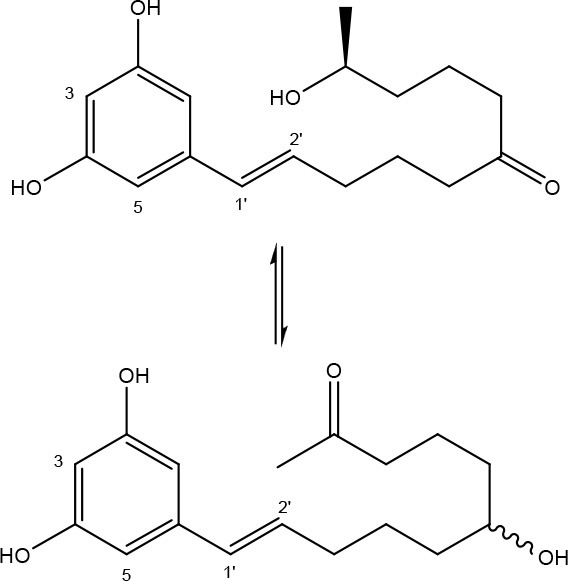	*Gliocladium roseum* RRL1859	El-sharkawy S. and Abul-hajj Y. J., 1988
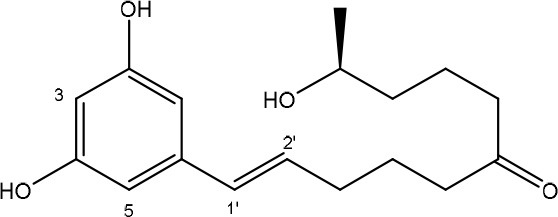	*Clonostachys rosea* IFO 7063 (a near-isogenic strain of NRRL 1859)	Kakeya et al., 2002; Takahashi-Ando et al., 2004
with decarboxylation		
No α-zearalenol and α-zearalanol observed, CO_2_-emmission indicative of decarboxylation	Culture extract of *B. natto* CICC 24640 and *B. subtilis* 168	Tinyiro et al., 2011
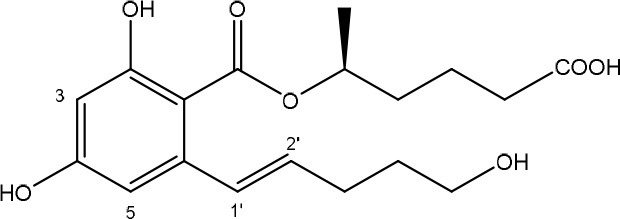	*Trichosporon mycotoxinivorans*	Molnar et al., 2004; Vekiru et al., 2010
No decarboxylation No α - nor β-zearalenol detected		
**CLEAVAGE OF THE AROMATIC RING**		
ZEN-A	*Aspergillus niger* strain FS10	Sun et al., 2014
ZEN-B: cleaved aromatic ring reduced liver and kidney damage (rats)		
ZEN-1 and ZEN-2: cleaved aromatic ring reduced estrogenic effects	*Acinetobacter* sp. SM04^*^	Yu et al., 2011a
**REDUCED TOXICITY CONFIRMED (NO DETOXIFICATION PRODUCTS IDENTIFIED)**		
Reduced toxicity of ZEN and α- and β- zearalenone to *Artemia salina*	*Pseudomonas* sp. ZEA-1^*^	Altalhi, 2007
Decrease or complete removal (K408) of estrogenic effects	*Rhodococcus* sp. *Rhodococcus pyridinivorans* K408	Kriszt et al., 2012; Cserháti et al., 2013; Krifaton et al., 2013
8′(S)-hydroxyzearalenone and 2,4-dimethoxyzearalenone: no binding to rat estrogen receptor	*Streptomyces rimosus*	El-sharkawy S. H. and Abul-hajj Y. J., 1988

* uses ZEN as sole carbon source.

A second cleavage pathway is exhibited by the yeast *Trichosporum mycotoxinivorans* (Molnar et al., 2004) to ZOM-1 intermediate (cleavage at the C6-ketone group), suggested to take place through a lactone intermediate and subsequent activity by unspecified a/b-hydrolase, but without the decarboxylation as seen in *C. rosea.* ZOM-1 did not show any estrogenic activity in a yeast bioassay, nor interaction with the human estrogen receptor (Vekiru et al., 2010), nor estrogenic activity with MCF-7 cells (Liu et al., 2001). It is important to notice that *T. mycotoxinivorans* is well-known in medicine, since it can cause opportunistic infections or induce summer-type hypersensitivity pneumonitis in immune-deficient cystic fibrosis patients (Tintelnot et al., 2011) which can be an impediment for applications.

Detoxification of ZEN contaminated corn steep liquor by *A. niger* strain FS10 and its culture filtrate, exemplified by less severe liver and kidney damage in rats, was recently reported (Sun et al., 2014). Two intermediate products, ZEN-A and ZEN-B, which inferred reduced liver and kidney damage in rats compared to ZEN, were detected, of which the latter the authors suggested the benzene ring might be cleaved because the UV absorption of ZEN was lost in ZEN-B. Somewhat similarly, two degradation products (ZEN-1 and ZEN-2) were detected after degradation by *Acinetobacter* sp. SM04 isolated from agricultural soil, for which no equally estrogenic activity could be detected on the basis of the MTT (tetrazolium salt) cell proliferation assay in MCF-7 cell line. Also, UV-Vis spectroscopy indicated cleavage of the benzene ring in these products (Yu et al., 2011a). Interestingly, ZEN and its estrogenic properties were only reduced when degradation tests were performed with extracellular extracts from M1 medium cultures, where sodium acetate is the only extra carbon source, and not from Nutrient Broth cultures, where many different extra carbon sources are present (Yu et al., 2011b). This indicates that the transcription of genes responsible for ZEN degradation may be regulated by catabolite repression.

*Pseudomonas* sp. ZEA-1, isolated from the rhizosphere of a corn plant, was shown to harbor the responsible degradation genes on a 120 kb plasmid mediating the transformation of ZEN and its derivatives α- and β- ZEN into less toxic products to *Artemia salina*. The transformation product was not elucidated, other than the specific absorption maximum at 400 nm (Altalhi, 2007). A 5.5 kb fragment containing the gene(s) encoding for ZEN degradation was cloned and actively expressed in *Escherichia coli* (Altalhi and El-Deeb, 2009).

The complete loss of ZEN estrogenic activity was obtained by several degrading *Rhodococcus* strains (Kriszt et al., 2012; Cserháti et al., 2013), without the identification of possible metabolites. *R. pyridinovorans* K408 showed a biodegradation potential of up to 85% and decreased the estrogenicity with 76%. Several strains also simultaneously degraded AFB1, ZEN, and T2-toxin (Cserháti et al., 2013), confirming the status *Rhodococcus* as a metabolically highly versatile genus with a large potential for degradation of aromatic and other pollutants (Larkin et al., 2005).

### Trichothecenes

#### Toxicity

Trichothecenes are sesquiterpenoids produced by mainly the genera *Fusarium, Trichothecium, Myrothecium, Trichoderma*, and *Stachybotrys* fungi (Sudakin, 2003; Kimura et al., 2007; Li et al., 2011). High doses lead to emesis, whereas low doses induce decreased feed consumption and weight gain (Eriksen and Pettersson, 2004). Trichothecenes are characterized by a 12,13-epoxy-trichothec-9-ene nucleus (Hussein and Brasel, 2001). Type A trichothecenes do not contain carbonyl function at C8 (T-2 toxin, HT-2 toxin, T-2 tetraol, T-2 triol, 15-monoacetoxyscirpenol, DAS, neosolaniol, and scirpentriol). Type B trichothecenes have a carbonyl group at C8 [deoxynivalenol (DON), 15-acetyl DON, 3-acetyl DON, nivalenol (NIV), 4-acetyl NIV]. Type C trichothecenes include another epoxide group and type D trichothecenes contains an additional ring system between C4 and C15 position (Zhou et al., 2008; McCormick et al., 2011).

The 12,13-epoxide ring in trichothecenes is essential for their toxicity (Zhou et al., 2008) and has been linked to the cytotoxicity of trichothecenes, namely inhibition of protein, RNA and DNA synthesis (Hussein and Brasel, 2001; Rocha et al., 2005). Trichothecenes bind with the 60S subunit of the ribosome and interfere with the action of peptidyltransferase (Ehrlich and Daigle, 1987). However, the degree of toxicity is dependent on the presence of substituents on C15 and C4 (Cundliffe et al., 1974; Cundliffe and Davies, 1977). The most potent mycotoxin T-2 toxin has acetyl or acyl side groups on C4, C8, and C15 of the basic structure. Loss of a side group from either of these positions resulted in reduced protein synthesis inhibition (T-2 toxin to HT-2 toxin, neosolaniol, or DAS). Further removal of side groups weakens their effect (T-2 triol, T-2 tetraol, 15-monoacetyl DAS, scirpentriol, fusarenon X, and DON) and reduction of hydroxyl groups, forming verrucarol, reduced their effectiveness greatly (Thompson and Wannemacher, 1986; Table 2 in Supplementary Material). De-acylation is clearly a first step toward detoxification, illustrated in Figure 1 in Supplementary Material. This reduced effect of de-acylation of T-2 toxin is also confirmed with human melanoma SK-Mel/27 cell lines (Babich and Borenfreund, 1991) and β-galactosidase activity of *Kluyveromyces marxianus* (Engler et al., 1999) showing the same tendency (Table 1 in Supplementary Material).

#### Degradation: organisms and pathways

The toxicity of trichothecenes is, next to their epoxide-group, also dependent on their acylated side chains. Therefore, two main groups are distinguished; acylated (e.g., T-2 toxin) and non-acylated trichothecenes (e.g., DON).

As previously described, de-acylation is the first step in detoxification of acylated trichothecenes. Degradation of T-2 toxin to HT-2 toxin and subsequently to T-2 triol was performed by *Curtobacterium* sp. strain 114-2 of which the reduced toxicity of T-2 triol was once more confirmed resulting in 23 and 13 times less toxic than, respectively T-2 toxin and HT-2 toxin (Ueno et al., 1983; Table 4). Still, the epoxide group in trichothecenes remains responsible for their toxicity. De-epoxidation is the next step of detoxification trichothecenes. Several studies focuses on the degradation of multiple trichothecenes and the differences between their metabolism by the same organism(s). Young et al. (2007) studied the metabolism of diverse trichothecenes by chicken intestinal microbes. For the non-acylated trichothecenes (4-DON, NIV, and verrucarol) their de-epoxidized metabolites were observed, for DAS, neosolaniol and T-2 toxin only de-acylation was exhibited and for the monoacetyl trichothecenes (3-acetyl DON, 15-acetyl DON, and fusarenon X), de-acylation was the predominant pathway. In another study, pig gastrointestinal microflora transformed 3-acetyl DON into DON and which was further de-epoxidized (Eriksen et al., 2002). Rat intestinal microflora was also able to de-epoxidize T-2 tetraol and scirpentriol, transform T-2 toxin into de-epoxy HT-2 toxin and de-epoxy T-2 triol and DAS into de-epoxymonoacetoxyscirpenol and de-epoxyscirpentriol (Swanson et al., 1987). All above mentioned cases concern degradation by mixed cultures. In contrast, *Eubacterium* BBSH 797 has the ability to degrade several trichothecenes as pure culture isolated from bovine rumen fluid (Fuchs et al., 2000, 2002; Binder and Binder, 2004) and has been developed into a commercial product (Biomin^®^ BBSH 797) for detoxifying trichothecenes in animal feed (He et al., 2010). It is known for its detoxification capacities of DON into DOM-1 and de-epoxidization of NIV, T-2 tetraol, scirpentriol, and HT-2 toxin. T-2 toxin was de-acylated into HT-2 toxin, whereas degradation of T-2 triol involved the competition of two reactions; (1) de-epoxidation or (2) deacylation into T-2 tetraol and subsequently de-epoxidation into de-epoxy T-2 tetraol (Fuchs et al., 2002). Further, 4-acetyl NIV and 3-acetyl NIV was de-acetylated and/or de-epoxidized (Fuchs et al., 2000).

**Table 4:** **Degradation and/or detoxification products of acylated trichothecenes**.

**Degradation and/or detoxification product**	**Microorganism**	**References**
**DEACETYLATION**		
T-2 toxin → HT-2 toxin	*Eubacterium* BBSH 797	Fuchs et al., 2002
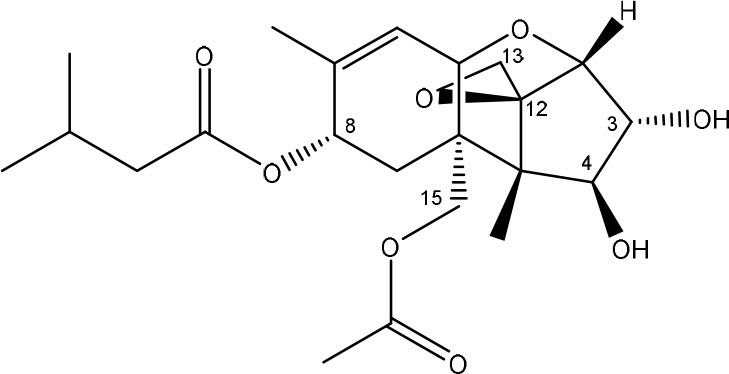	Carboxylesterase (from rat liver microsomes) (EC 3.1.1.1)	Ohta et al., 1977; Johnsen et al., 1986
(Figure: HT-2 toxin)		
T-2 toxin → HT-2 toxin → T-2 triol	*Curtobacterium* sp. strain 114-2	Ueno et al., 1983
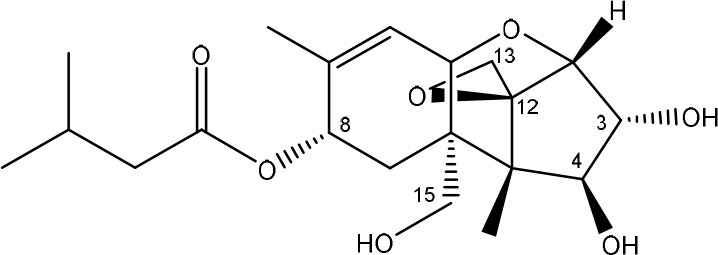	*Anaerovibrio lipolytica, Selenomonas ruminantium*	Westlake et al., 1987
	Bacterial community from soil or freshwater	Beeton and Bull, 1989
(Figure: T-2 triol)		
T-2 toxin → HT-2 toxin, T-2 triol, neosolaniol	*Butyrivibrio fibrisolvens* CE51	Westlake et al., 1987
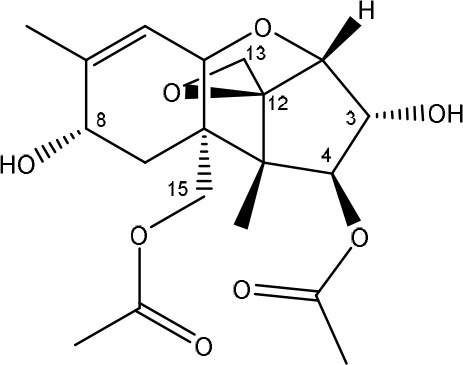		
(Figure: neosolaniol)		
T-2 toxin → neosolaniol	*Blastobotrys capitulata* strain	McCormick et al., 2012
Diacetoxyscirpenol → monoacetoxyscirpenol and scirpentriol	Fecal microflora from chickens, horses or dogs	Swanson et al., 1987
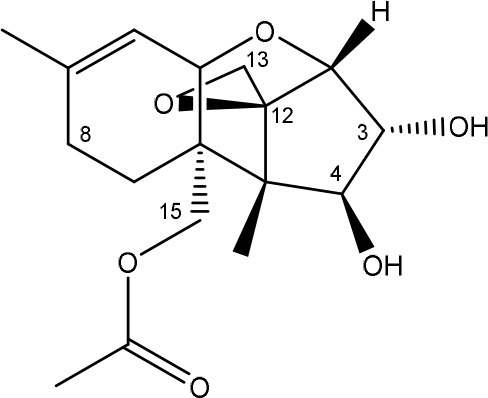		
(Figure: monoacetoxyscirpenol)		
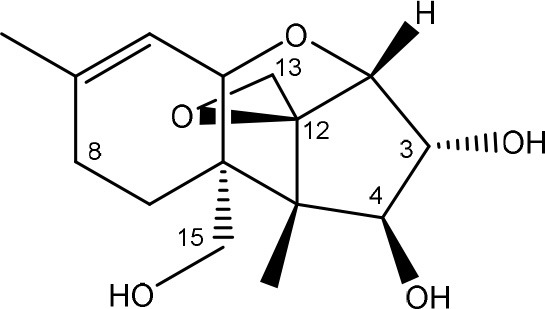		
(Figure: scirpentriol)		
DAS → monoacetoxyscirpenol	*Butyrivibrio fibrisolvens* M-14a (from ovine rumen fluid)	Matsushima et al., 1996
**DEACETYLATION AND DE-EPOXIDATION**		
Diacetoxyscirpenol → de-epoxymonoacetoxyscirpenol and de-epoxyscirpentriol	Mixed culture from intestinal microflora from rats	Swanson et al., 1987
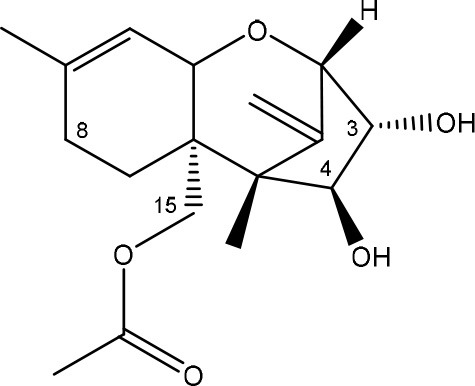		
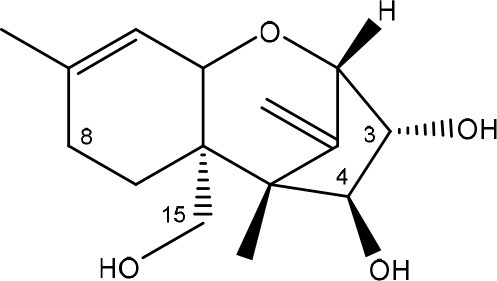		
T-2 triol → 1. de-epoxy; 2. T-2 tetraol → 3. de-epoxy T-2 tetraol	*Eubacterium* BBSH 797	Fuchs et al., 2002
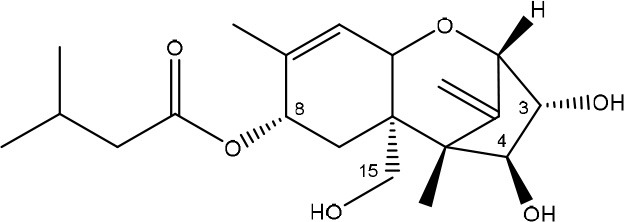		
1.		
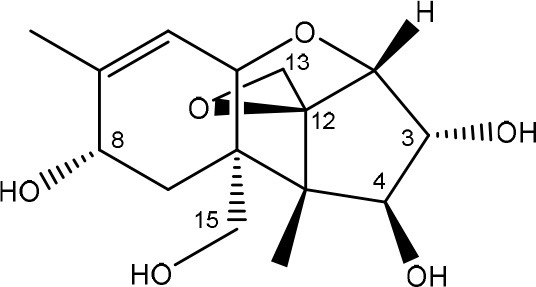		
2.		
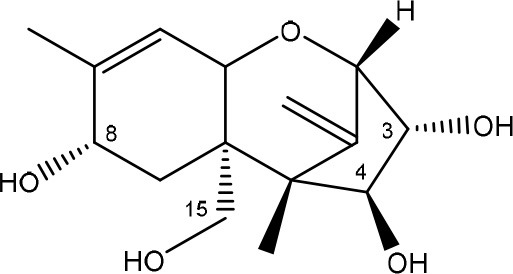		
3.		
**DE-EPOXIDATION**		
T-2 toxin → de-epoxy HT-2 toxin and de-epoxy T-2 triol	Mixed culture from intestinal microflora from rats	Swanson et al., 1987
HT-2 toxin → de-epoxy HT-2 toxin	*Eubacterium* BBSH 797	Fuchs et al., 2002
T-2 tetraol → de-epoxy T-2 tetraol	*Eubacterium* BBSH 797	Fuchs et al., 2002
**3-ACETYLATION**		
T-2 toxin → 3-acetyl T-2 toxin	*Blastobotrys parvus* strain	McCormick et al., 2012
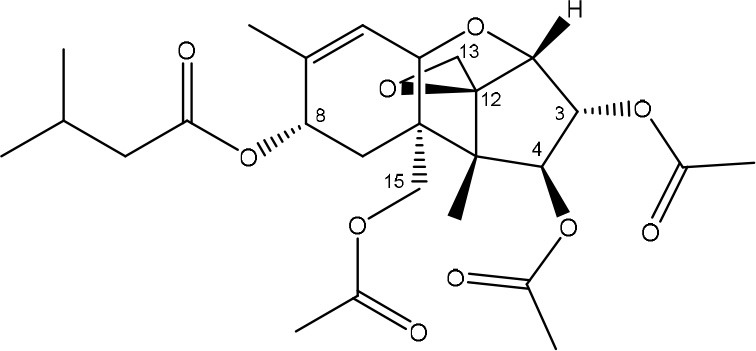		

Degradation of DON occurs through de-epoxidation, oxidation, or isomerization (Table 5). Microbial culture C133 of fish guts transformed DON to DOM-1 (Guan et al., 2009). *Eubacterium* BBSH 797 is known to degrade DON into DOM-1 anaerobically (Binder and Binder, 2004). *Citrobacter freundii* could transform DON into DOM-1 aerobically (Rafiqul, 2012). DON can also be oxidized to 3-keto DON which is 10 times less toxic than DON evaluated with a bioassay based on mitogen-induced and mitogen-free proliferations of mouse spleen lymphocytes (Shima et al., 1997). The bacterium strain E3-39 which degraded DON to 3-keto-DON, is belonging to the *Agrobacterium-Rhizobium* group. A mixed culture from environmental sources could degrade DON into 3-keto-DON, whereas 15-acetyl DON, 3-acetyl DON and fusarenon-X were also transformed (Volkl et al., 2004). Subsequently, He (2015) found the soil bacterium *Devosia mutans* 17-2-E-8 which transformed DON into 3-epi-DON (major product) and 3-keto-DON (minor product). These metabolites have also been tested on their toxicity with two assays. The IC_50_ values of 3-epi-DON and 3-keto-DON were 357 and 3 times higher, respectively, than that of DON on the basis of a MTT bioassay using Caco-2 cell line to asses cell viability, and were 1181 and 5 times higher, respectively, than that of DON on the basis of a cell proliferation BrdU bioassay using 3T3 fibroblast cell line to asses DNA synthesis (Table 1 in Supplementary Material). Toxicological effects of 14-day oral exposure of B6C3F_1_ mouse to DON and 3-epi-DON were also investigated concluding that 3-epi-DON was at least 50 times less toxic than DON (He, 2015). The metabolite 3-epi-DON was also formed by degradation of DON through *Nocardioides* sp. strain WSN05-2 isolated from a wheat field (Ikunaga et al., 2011). And lastly, nine *Nocardioides* strains (Gram-positive) and four *Devosia* strains (Gram-negative) produced 3-epi-DON aerobically. The Gram-positive strains showed DON assimilation, whereas the Gram-negatives did not (Sato et al., 2012).

**Table 5:** **Degradation and/or detoxification products of non-acylated tricothecenes**.

**Degradation and/or detoxification product**	**Microorganism**	**References**
**DE-EPOXIDATION**		
DON → de-epoxy DON (DOM-1)	*Eubacterium* BBSH 797 (anaerobically)	Binder and Binder, 2004
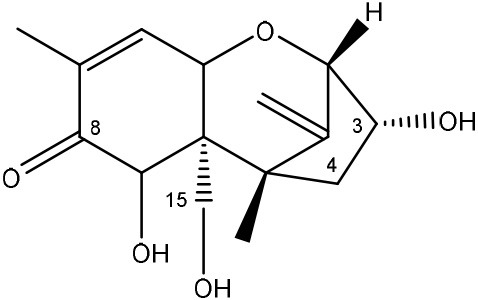	Chicken intestinal microbes	Young et al., 2007
	Isolate from chicken guts	Zhou et al., 2007
	Microbial community C133 (from fish guts)	Guan et al., 2009
	Isolate LS-100 (99% ~*Bacillus arbutinivorans*) (from chicken intestines)	Yu et al., 2010
	*Citrobacter freundii* (aerobically)	Rafiqul, 2012
nivalenol (NIV) → de-epoxy NIV	*Eubacterium* BBSH 797	Fuchs et al., 2000
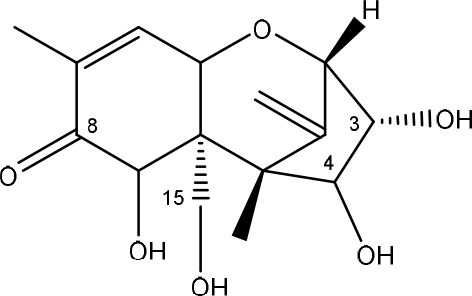	Chicken intestinal microbes	Young et al., 2007
	Microbial community C133 (from fish guts)	Guan et al., 2009
verrucarol → de-epoxy verrucarol	Chicken intestinal microbes	Young et al., 2007
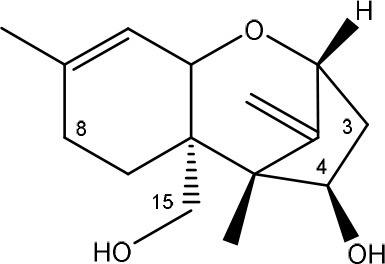		
	Microbial community C133 (from fish guts)	Guan et al., 2009
**C3 MODIFICATION THROUGH OXIDATION**		
3-keto-DON	*Agrobacterium-Rhizobium* strain E3-39 (soil)	Shima et al., 1997
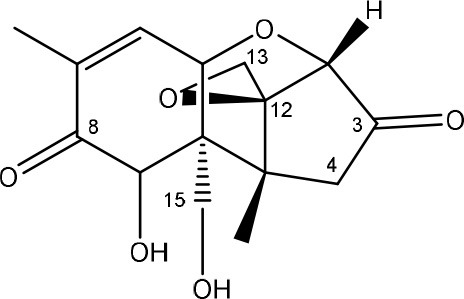	Mixed culture	Volkl et al., 2004
	*Devosia mutans* strain 17-2-E-8	He, 2015
**C3 MODIFICATION THROUGH EPIMERIZATION**		
3-epi-DON	*Nocardioides* strain WSN05-2 (soil, wheat field)^*^	Ikunaga et al., 2011
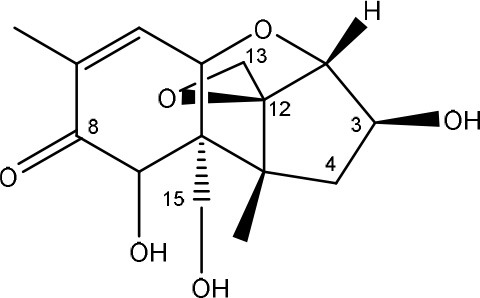	*Nocardioides* strains (environmental samples (field soils, wheat leaves))*	Sato et al., 2012
	*Devosia* strains (environmental samples (field soils, wheat leaves))	Sato et al., 2012
	*Devosia mutans* strain 17-2-E-8	He, 2015

* growth as sole carbon source.

Further, hydroxylation and glycosylation of trichothecenes are also known for their detoxification capability (He et al., 2010), however these derivatives can be rehydrolyzed or regenerated in the digestive tract of animals and humans losing their detoxification capacity.

### Aflatoxins

#### Toxicity

Aflatoxins are furanocoumarins produced by mainly *Aspergillus* species (Wu et al., 2009; Samuel et al., 2013). Naturally occurring aflatoxins are categorized by IARC as carcinogenic to humans (Group 1; IARC, 2002). Aflatoxin B1 (AFB1) is activated by cytochrome P450 system to a highly reactive AFB1-8,9-epoxide which can react with DNA (Eaton and Groopman, 1994; Guengerich et al., 1996).

The toxicity of AFB1 is mainly caused by the lactone ring. Cleavage of the lactone ring leads to a non-fluorescent compound with reduced biological activity (Lee et al., 1981). The residual component has a 450 times reduced mutagenicity (measured with the Ames test) and a 18 times reduced toxicity (measured with chicken embryo test; Lee et al., 1981). Also the difuran ring moiety, especially the presence of the double bond in the terminal furan ring, contributes to the toxicity (Wogan et al., 1971) as evidenced by comparing the toxicity of aflatoxins with similar coumarin molecules. Wong and Hsieh (1976) concluded by comparing several aflatoxins and metabolites with the Ames test that the double bond was also involved in both mutagenic and carcinogenic activity of aflatoxins leading that the aflatoxins AFB2 and AFG2 (without a double bond) are much less toxic than AFB1 and AFG1 (with a double bond).

#### Degradation: organisms and pathways

To our knowledge, a first report on the microbial detoxification of AFB1 has been published in 1966, mediated by *Flavobacterium aurantiacum* (now called *Nocardia corynebacterioides*; Ciegler et al., 1966; Teniola et al., 2005). Although no detoxification products were analyzed, residual toxicity to ducklings was found to be absent indicating true detoxification (Ciegler et al., 1966). The biosafety of the microorganism was confirmed using an *in vivo* trial with chickens (Tejada-Castañeda et al., 2008). Since this first report, many studies have focused on the detoxification of AFB1. However, only a few studies detected the degradation products and analyzed their toxicity. Generally, two main detoxification pathways are observed: modification of the difuran ring or modification of the coumarin structure.

Firstly, modification of the difuran ring moiety was reported in several studies. Degradation of AFB1 into AFB1-8,9-dihydrodiol was performed by manganese peroxidase from the white rot fungi *Phanerochaete sordida* (Wang et al., 2011) and the “aflatoxin-detoxifizyme (ADTZ)” of fungus *Armillariella tabescens* (Liu et al., 1998b; Table 6). The authors suggested that AFB1 degradation initially involves formation of AFB1-8,9-epoxide, after which a hydrolysis resulted in a dihydrodiol-derivate. Detoxification was confirmed with a reduced mutagenicity measured by the Ames *Salmonella*-based test (Liu et al., 1998b, 2001; Wang et al., 2011; Table 1 in Supplementary Material) and reduced toxicity measured with rat liver (Liu et al., 1998b) and chicken embryos (Liu et al., 1998a). Another metabolite was detected with the white rot fungus *Pleurotus ostreatus* GHBBF10 which degraded 91.76% of AFB1 into a component which could be a hydrolyte of AFB1, namely dihydrohydroxyaflatoxin B1 (AFB2a) (Das et al., 2014; Table 2). AFB2a has also a reduced mutagenicity (Wong and Hsieh, 1976; Table 1 in Supplementary Material).

**Table 6:** **Degradation and/or detoxification products of aflatoxins**.

**Degradation and/or detoxification product**	**Microorganism**	**References**
**AFB1-8,9-DIHYDRODIOL**		
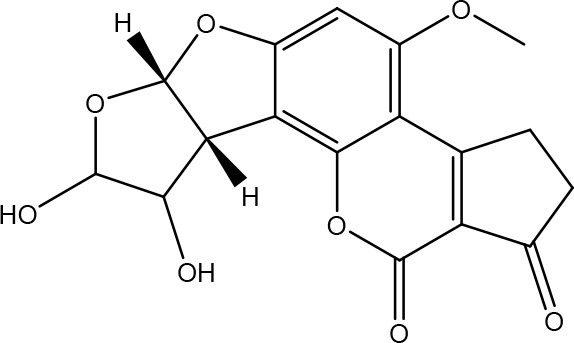	*Armillariella tabescens*	Liu et al., 1998a,b
	*Phanerochaete sordida*	Wang et al., 2011
**AFB2a**		
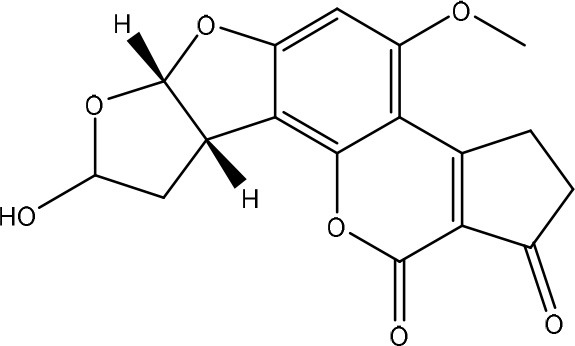	*Pleurotus ostreatus* GHBBF10	Das et al., 2014
		Wong and Hsieh, 1976
**AFD1**		
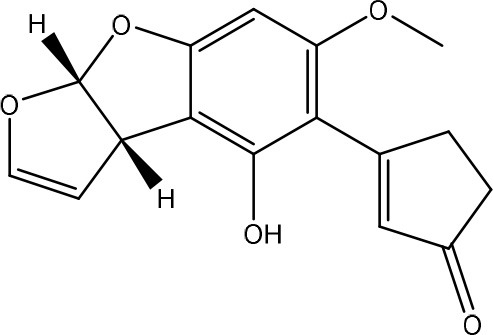	*Pseudomonas putida*	Samuel et al., 2014
		Grove et al., 1984
**AFD2**		
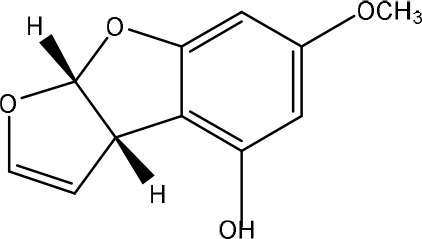	*Pseudomonas putida*	Samuel et al., 2014
**AFD3**		
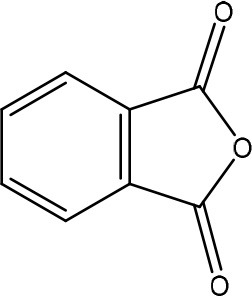	*Pseudomonas putida*	Samuel et al., 2014
**REDUCED TOXICITY CONFIRMED (NO DETOXIFICATION PRODUCTS IDENTIFIED)**		
reduced mutagenicity	*Flavobacterium aurantiacum (= Nocardia corynebacterioides)*	Ciegler et al., 1966; Teniola et al., 2005
	laccase enzyme (*T. versicolor)* recombinant laccase enzyme *(A. niger)*	Alberts et al., 2009
	*Rhodococcus erythropolis*	Alberts et al., 2006
reduced genotoxicity with SOS-chromotest	several *Rhodococcus* sp.	Cserháti et al., 2013
reduced toxicity in *Aliivibrio fischeri* or with SOS-chromotest	several *Rhodococcus* sp. and *Pseudomonas* sp.	Krifaton et al., 2011

Secondly, the lactone ring in the coumarin moiety of AFB1 can be changed. A *Pseudomonas putida* strain has been discovered degrading AFB1 into AFD1 and subsequently into AFD2 (Table 6). The metabolite AFD1 had been previously discovered through ammonization and acidifying AFB1, whereas the difuran ring stays unchanged and the lactone ring is cleaved. AFD1 has a lower mutagenicity and toxicity measured by respectively the Ames *Salmonella*-based test (Méndez-Albores et al., 2005) and HeLa cells with the MTT [3-(4,5-dimethylthiazole-2-yl)-2,5-diphenyltetrazolium bromide] method (Samuel et al., 2014). The metabolites AFD2 (an aflatoxin metabolite lacking the lactone and cyclopentenone ring) and AFD3 also showed a lower toxicity toward HeLa cells (Table 1 in Supplementary Material; Samuel et al., 2014).

In certain studies, no degradation product was identified, but toxicity tests were performed on the treated AFB1. Similarly to *F. aurantiacum* as mentioned before, a pure laccase enzyme from *Trametes versicolor* and a recombinant laccase enzyme produced by *A. niger* degraded, respectively, 87.34 and 55% of AFB1 with a significant loss of mutagenicity evaluated in the Ames *Salmonella*-based assay (Alberts et al., 2009). Extracellular enzymes of *Rhodococcus eryhtropolis* were also able to detoxify AFB1 with a loss of mutagenicity (Alberts et al., 2006).

### Ochratoxins

#### Toxicity

Ochratoxins (OT) are a group of mycotoxins sharing an isocoumarin moiety substituted with a phenylalanine group (OTA, OTB, hydroxyl-OTA), a phenylalanine ester group (OTC, OTA methylester, OTB methyl ester, OTB ethyl ester), or a hydroxyl group (OTα and OTβ). OTA is the most important OT because of its incidence in food- and feed commodities. It is composed of a 7-carboxy-5-chloro-8-hydroxy-3,4-dihydro-3-R-methylisocoumarin (OTα) moiety and the amino acid L- phenylalanine group. Both structures are linked through a carboxy group via an amide bond. OTA is produced by *Aspergillus* and *Penicillium* species (Richard, 2007; McCormick, 2013). The mode of action of OTA is broad and therefore the molecule has nephrotoxic, mutagenic, teratogenic, neurotoxic, hepatotoxic, and immunotoxic properties (Pfohl-Leszkowicz and Manderville, 2007). The toxicity of OTA is mainly attributed to its isocoumarin moiety and probably not to the phenylalanine moiety (Table 1; Xiao et al., 1996). The carboxyl group of the phenylalanine moiety and also the Cl group of the other moiety seem to be conducive for the toxicity of OTA.

#### Degradation: organisms and pathways

The main detoxification pathway of OTA is the hydrolyzation of the amide bond between the isocoumarin residue and phenylalanine by a carboxypeptidase. Two classes of carboxypeptidases have been associated with degradation of OTA namely Carboxypeptidase A (CPA) (Stander et al., 2001; Chang et al., 2015) and Y (CPY) (Dridi et al., 2015). The main difference between both is the use of a zinc ion within the protein for hydrolysis of the peptide at the C-terminal of the amino acid. Almost all strains that are reported to degrade OTA use this pathway resulting in the formation of L-β- phenylalanine and OTα the former being less toxic than OTA (Table 7; Bruinink and Sidler, 1997). Although this is a very straightforward way of reducing the amount of OTA in food and feed samples, it is important to highlight that the efficient degradation of OTA is depending on the activity of the peptidase enzyme. With this respect, several research groups showed that these carboxypeptidase enzymes tend to have high optimal temperatures (30°C or higher) which might hamper practical applications, observed with *Pediococcus parvulus* and several yeasts such as *Pfaffia rhodozyma* (Péteri et al., 2007; Patharajan et al., 2011; Abrunhosa et al., 2014). Other enzymes are also able to carry out this reaction: Deoxygenases, lipases, amidases, and several commercial proteases (Abrunhosa et al., 2006), have also been identified as carrying out this reaction. Although depending on the enzyme, intermediates can be different, the end product is always OTα.

Some interesting strains are highlighted here. *Trichosporon mycotoxinivorans* was demonstrated to deactivate OTA by conversion into the nontoxic OTα. Even more intriguingly, *T. mycotoxinivorans* was also able to decarboxylate ZEN (Molnar et al., 2004; Vekiru et al., 2010). After 24 h, ZEN was degraded to carbon dioxide or into metabolites that neither showed fluorescence nor did absorb UV-light. Neither α- nor β-ZEL, other equally estrogenic metabolites of ZEN, could be detected. It is commercially applied as feed additive under the commercial name Biomin^®;^ MTV.

**Table 7:** **Degradation and/or detoxification products of ochratoxins**.

**Degradation/detoxification product**	**Microorganism**	**References**
**ENZYMATIC REMOVAL OF PHENYLALANINE GROUP**	**BACTERIA**	
ochratoxin α	*Bacillus licheniformis*	Petchkongkaew et al., 2008
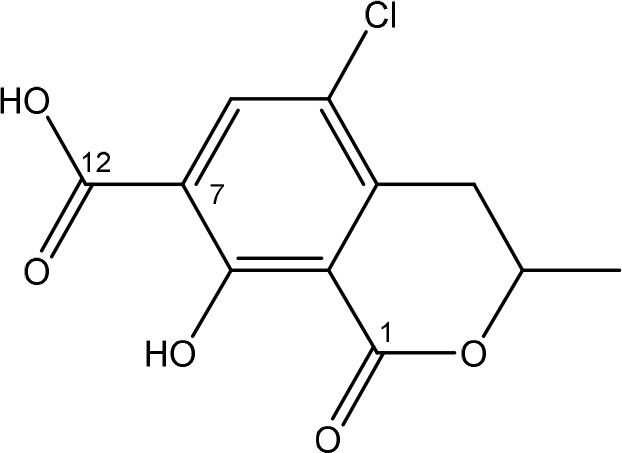	*Bacillus* spp.	
	*Brevibacterium linens*	Rodriguez et al., 2011
	*Brevibacterium iodinum*	
	*Brevibacterium epidermidis*	
	*Acinetobacter calcoaceticus*	Hwang and Draughon, 1994; De Bellis et al., 2015
	*Penylobacterium immobile*	Wegst and Lingens, 1983
	*Bacillus amyloliquefaciens ASAG1*	Chang et al., 2015
	*Cupriavidus basilensis Or16*	Ferenczi et al., 2014
	*Pediococcus parvulus*	Abrunhosa et al., 2014
	*Lactobacillus acidophilus*	Fuchs et al., 2008
**ENZYMATIC REMOVAL OF PHENYLALANINE GROUP**	**FUNGI**	
ochratoxin α	*Aspergillus clavatus*	Abrunhosa et al., 2002; Bejaoui et al., 2006
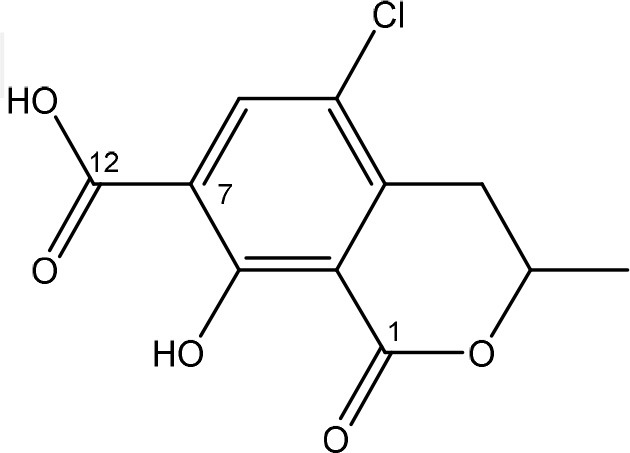	*Aspergillus vesicolor*	
	*Aspergillus niger*	
	*Aspergillus japonicas*	
	*Aspergillus alliaceus*	
	*Aspergillus alliaceus*	
	*Aspergillus ochraceus*	
	*Aspergillus wentii*	
	*Trichosporon mycotoxinivorans*	Molnar et al., 2004
	*Botrytis spp*	Abrunhosa et al., 2002
	*Alternaria* spp.	
	*Penicillium* spp.	
	*Cladosporium* spp.	
	*Rhizopus spp*	Varga et al., 2005
	*Pleurotus ostreatus*	Engelhardt, 2002
	*Saccharomyces* spp.	
	*Rhodoturula* spp.	
	*Cryptococcus* spp.	
	*Pfaffia rhodozyma*	Péteri et al., 2007
	*Aureobasidium pullulans*	de Felice et al., 2008
	*Aspergillus niger*	Dobritzsch et al., 2014
	*Aspergillus niger*	Stander et al., 2000

*Phenylobacterium immobile* (Wegst and Lingens, 1983) was also found to convert OTA to OTα through a dioxygenase step on the phenylalanine moiety, a dehydrogenation to catechol, a ring cleavage, and the final formation of OTα via a hydrolase.

## Unexplored worlds that might harbor valuable mycotoxin degrading microorganisms

### Targeting carboxyl esters

As stated above, fumonisins and acylated trichothecenes share carboxyl-esters which are involved in their toxicity. Detoxification of fumonisins is realized by removal of the tricarballylate side groups via carboxylesterases (EC 3.1.1.1). Similarly, acylated trichothecenes have several side groups where carboxylesterases could attack on the carboxyl group, as observed with carboxylesterases from rat liver microsomes (categorized as EC 3.1.1.1) degrading T-2 toxin into HT-2 toxin (Ohta et al., 1977; Johnsen et al., 1986). Carboxylesterases are multifunctional enzymes that catalyze the hydrolysis of substrates containing ester, amide, and thioester bonds with relatively broad substrate specificity (Bornscheuer, 2002) which is attributed to a large conformable active site that permits entry of numerous structurally diverse substrates. Microbial carboxylesterases have been reported in the degradation of pesticides; some hydrolyze pyrethroids and bind stoichiometrically to carbamates and organophosphates reviewed by Singh (2014). Several organisms have been isolated which degradative capacities of these compounds inferred by the expression of carboxylesterases, for which, bearing in mind their general broad substrate specificity, it might be worthwhile to screen for degradation of fumonisins and acylated trichothecenes. For example, broad-spectrum pyrethroid-hydrolyzing carboxylesterases were identified in the lambda-cyhalothrin degrading *Ochrobactrum anthropic* YZ-1 strain (Zhai et al., 2012) and *Bacillus* sp. DG-02, isolated from a pyrethroid-manufacturing wastewater treatment system (Chen et al., 2014). Similarly, an *Acinetobacter baumannii* strain was shown to degrade a wide range of organophosphorus compounds and evidence for a novel carboxylesterase in this strain was presented. Taking an environmental DNA (eDNA) isolation approach, Rashamuse et al. (2009) screened a microbial community to access novel carboxylesterases from environmental genomes: a carboxylesterase gene with 60% sequence identity to the gene from *Ralstonia eutropha* was identified, along with subsequent heterologous expression in *Escherichia coli* in a biologically active form. A similar approach might be taken to discover more mycotoxin-active carboxylases based on sequences of carboxylases present in *Sphingomonas* sp. ATCC 55552, *Exophiala* sp., or *Sphingopyxis* sp. MTA 144.

### Targeting a lactone ring

The presence of a lactone moiety is shared by OTA, aflatoxins, and ZEN. Lactone chemicals are well-known as auto-regulators in both eukaryotic and prokaryotic cells. A well-known example is acyl homoserine lacton which is a quorum sensing molecule associated with biofilm formation. Because of the detrimental effects of biofilms in many industrial applications, high throughput research initiatives have been undertaken in the past and present in search for enzymes able to degrade these lactone molecules. These, often metagenomics, approaches result in the characterization of new and more efficient lactonase enzymes (Shimizu et al., 2001; Riaz et al., 2008; Schipper et al., 2009). The potential activities of these lactonases with respect to mycotoxins remains elusive but scientific fields studying biofilm issues might offer new microbial consortia ready to be explored for their mycotoxin degrading capacities.

Also targeted analyses can result in the characterization of new and efficient lactonase enzymes. In a screening assay for enzymes able to degrade bio-active lactones, a novel lactonohydrolase, an enzyme that catalyzes the hydrolysis of aldonate lactones to the corresponding aldonic acids, was purified from *Fusarium oxysporum* AKU 3702. The enzyme irreversibly hydrolyzes a broad spectrum of aromatic lactones, such as dihydrocoumarin and homogentisic-acid lactone (Shimizu et al., 1992; Kobayashi et al., 1998).

New insights for biodegradation of mycotoxins with estrogenic effects such as ZEN might come from studies on the microbial degradation of steroidal estrogens. Several strains have been isolated which are able to degrade the steroidal estrogen estrone (E1), also harboring a lactone ring (Yu et al., 2013), among which *Sphingomonas* sp. KC8 (Yu et al., 2007), *Bacillus subtilis* E2Y4 (Jiang et al., 2010), and several *Rhodococcus* sp. (Yoshimoto et al., 2004), remarkably all isolated from activated sludge. Also, cometabolic degradation of ethinyl estradiol (EE2) was obtained with nitrifying activated sludge (Vader et al., 2000). Therefore, activated sludge might prove to be a rich source of degradation potential for lactone-harboring mycotoxins.

### Targeting an epoxide moiety

For trichothecenes, the epoxide moiety is an important chemical group associated with toxicity. Microbial transformation of epoxides was studied by Swaving and de Bont (1998) who demonstrated that two types of enzymes were responsible for detoxification of epoxides: glutathione transferases as a class of general detoxifying enzymes and epoxide hydrolases which are specific for detoxification of epoxides. Glutathione transferases (dependent on glutathione as cofactor) are mostly found in aerobic eukaryotes and prokaryotes, such as *E. coli* and *Rhodococcus* sp. which degrades a range of epoxides. Epoxide hydrolases are found in many microorganisms, like *Flavobacterium, Pseudomonas, Corynebacterium*, and *Stigmatella* species. Other enzymes can also convert an epoxide intermediate via a certain pathway (e.g., alpha-pinene oxide lyase from *Nocardia* sp. strain P18.3 and *Pseudomonas fluorescens* NCIMB 11671, styrene oxide isomerase of *Pseudomonas* species, *Xanthobacter* 124X or *Exophilia jeanselmei*, or epoxyalkane-degrading enzyme in *Xanthobacter* Py2 (Swaving and de Bont, 1998). Broudiscou et al. (2007) proved that mono-and sesquiterpenes were degraded in the presence of mixed rumen microorganisms, corresponding with the isolation origin mostly found for microorganisms degrading trichothecenes. Ptaquiloside, also a sesquiterpene toxin, could be degraded by soil microorganisms (Engel et al., 2007) which can be a new source for biodegradation of trichothecenes.

### Targeting poly-aromatic ring structures

White rot fungi are frequently found for degrading aflatoxins, such as *A. tabescens, P. sordida, P. ostreatus, T. versicolor*, and *Peniophora* sp. (Liu et al., 1998b; Motomura et al., 2003; Alberts et al., 2009; Wang et al., 2011; Das et al., 2014; Yehia, 2014). White rot fungi are well-known for their degrading capabilities of their natural substrate lignin and a broad spectrum of structurally diverse toxic environmental pollutants (e.g., munitions waste, pesticides, polychlorinated biphenyls, polycyclic aromatic hydrocarbons, bleach plant effluent, synthetic dyes, synthetic polymers, and wood preservatives; Reddy, 1995; Pointing, 2001). Lignin peroxidases, manganese peroxidases and laccases are the major enzymes involved in lignin degradation based on oxidative mechanisms (Tuor et al., 1995). Laccases and manganese peroxidases of white rot fungi have been reported for degrading aflatoxins which possible can lead to different metabolites (Motomura et al., 2003; Wang et al., 2011). *Peniophora* sp. SCC0152, *P. ostreatus* St2-3, and several *Trametes* sp. strains demonstrated the degradation of Poly R-478 dye and AFB1 (Alberts et al., 2009). Next to white rot fungi, the genus *Rhodococcus* is also known to have promising degradation capability for xenobiotics (Martínková et al., 2009). Alberts et al. (2006) and Eshelli et al. (2015) suggested that degradation of AFB1 (polyaromatic compound) by a *Rhodococcus erythropolis* strain could be degraded in a similar way of degrading polyaromatic compounds of which their degradation occurs through a cascade of enzyme reactions (e.g., ring cleavage biphenyl dioxygenases, dihydrodiol dehydrogenases, and hydrolases). Degradation of a wide range of aromatic compounds results in a limited number of central intermediates (catechol, protocatechuate, gentisate) which are further degraded through central pathways for finally entering the citrate cycle (Martínková et al., 2009). In addition, *R. erythropolis* NI1 strain was found which was capable of degrading AFB1, ZEN, and T-2 toxin at the same time (Cserháti et al., 2013). Hence, various organisms have the potential for degrading multiple mycotoxins or other components, exemplified by. *Stenotrophomonas maltophilia, Stenotrophomonas* sp. NMO-3, and *Pseudomonas aeruginosa* which can degrade AFB1 and coumarin (Guan et al., 2008; Liang et al., 2008; Sangare et al., 2015), and *Mycobacterium fluoranthenivorans* FA4T which can degrade AFB1 and also grow on the polycyclic aromatic hydrocarbon fluoranthene (Hormisch et al., 2004).

Supporting the notion that microorganisms are able to metabolize structurally comparable chemicals from vastly different origins, a mixed enrichment culture capable of removing ZEN as sole carbon source, without the presence of derivatives, was obtained from soil collected at a coal gasification site, which are generally known to be associated with polycyclic aromatic hydrocarbon contamination. Removal of ZEN was enhanced in the presence of phenanthrene through enhanced microbial growth, indicating that organisms capable of using ZEN were also able to metabolize phenanthrene (Megharaj et al., 1997). Building further on this notion, cleaving the aromatic ring of ZEN by *A. niger* FS10 (Sun et al., 2014) and *Acinetobacter* sp. *SM04* (Yu et al., 2011a), for which no enzymes have been identified, might bear resemblance to the degradation of resorcinol (1,3-dihydroxybenzene) for which degradation is known by *P. putida* (Chapman Ribbons and Ribbons, 1976) and *Azotobacter vinelandii* (Groseclose and Ribbons, 1981).

### Targeting a carboxyl/amide moiety

Carboxypeptidase A and Y belong to the group of protease enzymes. Great interest in these enzymes comes from the field of wastewater treatments as these enzymes play a vital role in the extracellular catabolism of organic matter in activated sludge. In search of these enzymes, progressively more culture independent screening approaches are being employed as up to 90% of bacteria present in wastewater cannot be cultured and in this way a large reservoir of enzymes is overlooked. In matrices harboring a vast set of microorganisms that cannot be cultured, metagenomics analyses are often the solution to get an in depth insight into the complexity of these enzymes in a certain matrix. Pursuing this approach, a metagenomics analysis of waste water revealed a highly diverse phylogenetic diversity of carboxypeptidase gene sequences including previously undescribed types of carboxypeptidases which might be interesting to be applied for diverse biotechnological applications such as the remediation of OTA contaminated batches (Jin et al., 2014).

## Perspectives

Although fumonisins, trichothecenes, ZEN, OTA, and aflatoxins comprise the major mycotoxin groups in food- and feed commodities, there are several other mycotoxins that were not addressed in present review because knowledge on biodegradation and detoxification is scarce. Cyclodepsipeptides such as beauvericin and enniatins are increasingly reported in many countries in several commodities. However, to our knowledge, no reports are available on their biodegradation and detoxification by microorganisms.The same accounts for ergot alkaloids such as lysergic acid, ergine, and ergopeptines. They occur widely but to date, only one paper has recently reported on a *R. erythropolis* isolate able to degrade these compounds (Thamhesl et al., 2015). Finally, for the *Penicllium expansum* mycotoxin patulin, recent papers report on biodegradation of this mycotoxin by *Pichia caribbica* (Cao et al., 2013), *Metschnikowia pulcherrima* (Reddy et al., 2011), *Kodameae ohmeri* (Dong et al., 2015), *Rhodosporidium* spp. (Castoria et al., 2011; Zhu et al., 2015), and *Saccharomyces cerevisiae* (Moss and Long, 2002). Nevertheless, for these emerging and also for the other mycotoxins, there is still a considerable need for concerted research initiatives to identify new high-performance strains which can be implemented in practice.

Many surveys around the globe illustrate that mycotoxin contaminated batches of food and feed products often contain multiple both structurally related and non-related mycotoxins. An emerging approach to tackle this issue is biodegradation of mycotoxins by microorganisms. In our opinion an ideal biodegrading and detoxification agent should meet following features: (i) a fast and efficient degradation, (ii) of a broad spectrum of toxins, (iii) into non-toxic end products, (iv) by a non-pathogenic strain or consortium (v) under conditions that are relevant for the matrix in which the mycotoxin problem occurs. In order to do so, we urge researchers to look beyond the disappearance of the mother compound to rule out the creation of any *lesser evils*, and to *explore strange new worlds, seek out new organisms and new metabolic pathways*.

## Author contributions

KA and LD conceived the idea and scope for the review. IV, KA, and LD all equally contributed to gathering and summarizing the literature, designing the tables and figures, and writing and editing of the paper.

### Conflict of interest statement

The authors declare that the research was conducted in the absence of any commercial or financial relationships that could be construed as a potential conflict of interest.

## Supplementary material

Figure 1 **Reduced toxicity of T-2 toxin by subsequent de-acylation**.

Table 1 **Toxicity data on mycotoxins and their metabolites**.

Table 2 **Toxicity of trichothecenes (from most potent to almost no effectiveness observed) (Thompson and Wannemacher, 1986)**.
