# Supplementary figures and images for: Biodegradation of Mycotoxins: Tales from Known and Unexplored Worlds
Source: Front Microbiol. 2016 Apr 25;7:561. doi: 10.3389/fmicb.2016.00561 (PMC4843849; doi:10.3389/fmicb.2016.00561)

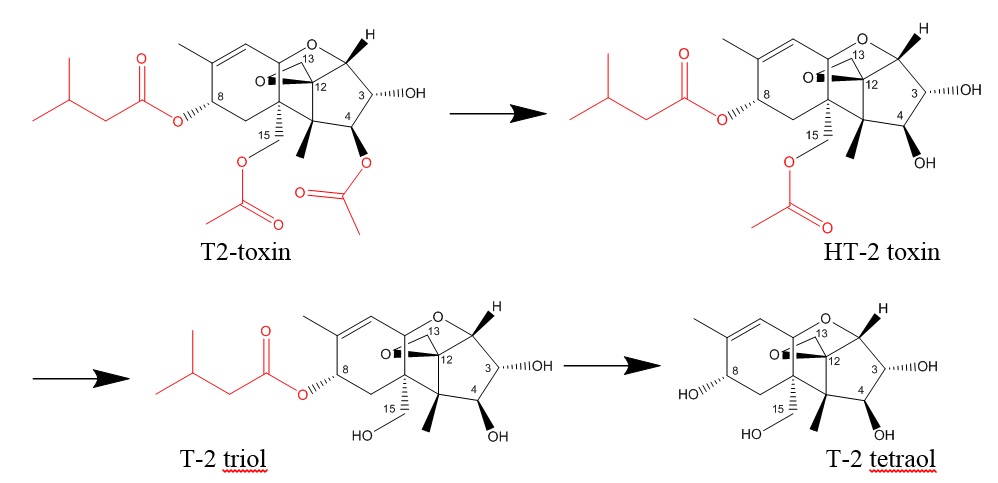

Supplement: Figure 1 — Reduced toxicity of T-2 toxin by subsequent de-acylation. [file Figure1.JPEG]
