# Supplementary material for: Biodegradation of Mycotoxins: Tales from Known and Unexplored Worlds
Source: Front Microbiol. 2016 Apr 25;7:561. doi: 10.3389/fmicb.2016.00561 (PMC4843849; doi:10.3389/fmicb.2016.00561)
Supplement: Table 1 — Toxicity data on mycotoxins and their metabolites. [file Table1.docx]

Table 8: Toxicity data on mycotoxins and their metabolites

| Fumonisins | |  | | | | | | References | | | |
| --- | --- | --- | --- | --- | --- | --- | --- | --- | --- | --- | --- |
|  | | IC50 (µM)  Canine kidney cells | IC50 (µM)  Rat hepatoma cells | | | IC50 (µM)  Mouse embryo fibroblasts | | (Abbas et al., 1995) | | | |
| FB1 | | 10 | 15 | | | 200 | |  | | | |
| AP1 (no TCA groups) | | 100 | 100 | | | 200 | |  | | | |
| N-acetylated AP1 (no TCA groups and inactive NH_2_-group) | | 300 | 150 | | | 150 | |  | | | |
| Zearalenone | |  | |  | | |  |  | | | |
| ZEN | | Similar estrogenic activity in human breast cancer MCF-7 compared to 17 β-estradiol | | | | | | (Kakeya et al., 2002) | | | |
|  | | No estrogenic activity in human breast cancer MCF-7 | | | | | |  |  |  |  |
|  | | Samples after 24 and 48 hours were non estrogenic to MCF-cells | | | | | | (Schatzmayr et al., 2003) | | | |
| Trichothecenes | |  | |  | | |  |  | | | |
|  | | EC_50_-value (µg/ml)  for inhibition of β-galactosidase activity of *Kluyveromyces marxianus* | | | | | | (Engler et al., 1999) | | | |
| T-2 | | 0,012 | | | | | |  | | | |
| diacetoxyscirpenol | | 0,03 | | | | | |  |  |  |  |
| HT-2 | | 0,07 | | | | | |  |  |  |  |
| neosolaniol | | 2,0 | | | | | |  |  |  |  |
| T-2 triol | | 5,0 | | | | | |  |  |  |  |
| scirpentriol | | 6,0 | | | | | |  |  |  |  |
| nivalenol | | 14 | | | | | |  |  |  |  |
| deoxynivalenol | | 21 | | | | | |  |  |  |  |
|  | | IC_50_ (nM)  in 5-bromo-2′-deoxyuridine (BrdU) incorporation assay in 3T3 mouse fibroblasts | | | | | | (Eriksen et al., 2004) | | | |
| DON | | 1,50 ± 0,34 | | | | | |  | | | |
| de-epoxy DON | | 83,0 ± 8,77 | | | | | |  | | | |
| NIV | | 1,19 ± 0,06 | | | | | |  | | | |
| de-epoxy-NIV | | 64,2 ± 3,14 | | | | | |  | | | |
|  | | IC50 (µg/ml) in MTT bioassay in Caco-2 cells | | | IC50 (µg/ml)  in BrdU bioassay in 3T3 mouse fibroblasts | | | (He, 2015) | | | |
| DON | | 0,409 | | | 0,238 | | |  | | | |
| 3-epi-DON | | 146 | | | 281 | | |  | | | |
| 3-keto-DON | | 1,24 | | | 1,08 | | |  | | | |
| Aflatoxin |  | | |  | | |  | | | |  |
|  | Mutagenicity: N° of revertants/plate in *Salmonella typhimurium* TA98 + rat liver S9 mix | | | Mutagenicity: N° of revertants/µg in *Salmonella typhimurium* TA98 + rat liver S9 mix | | | EC_50_ (µg/ml)  HeLa cells | |  | | |
| AFB1 | 966 ± 110 | | | 8527 ± 1434 | | | 2 | |  | | |
| AFB1-8,9-dihydrodiol | 56 ± 10 | | |  | | |  | | (Liu et al., 2001) | | |
| AFB2a |  | | | 2 ± 2 | | |  | | (Wong and Hsieh, 1976) | | |
| AFD1 |  | | |  | | | ˃10 | | (Samuel et al., 2014) | | |
| AFD2 |  | | |  | | | 5,2 | | (Samuel et al., 2014) | | |
| AFD3 |  | | |  | | | 7,5 | | (Samuel et al., 2014) | | |
|  |  | | |  | | |  | |  | | |
| Ochratoxin A | |  | |  | | |  |  | | | |
|  | | LC_50_ (nM)  HeLa cells | | | Minimum Toxic Dose (nmol/dics)  *Bacillus brevis* | | | | | (Xiao et al., 1996) | |
| ochratoxin A | | 0,005 | | | 8,7 ± 1,7 | | | | |  | |
| ochratoxin α | | 0,56 | | | 390 ± 20 | | | | |  | |
