# Supplementary material for: Biodegradation of Mycotoxins: Tales from Known and Unexplored Worlds
Source: Front Microbiol. 2016 Apr 25;7:561. doi: 10.3389/fmicb.2016.00561 (PMC4843849; doi:10.3389/fmicb.2016.00561)
Supplement: Table 2 — Toxicity of trichothecenes (from most potent to almost no effectiveness observed) (Thompson and Wannemacher, 1986). [file Table2.docx]

Table 9. Toxicity of trichothecenes (from most potent to almost no effectiveness observed) (Thompson and Wannemacher, 1986)

| **Most potent** | **Reduced effect** | **Weakened effect** | **Greatly reduced** |
| --- | --- | --- | --- |
|  T-2 toxin |   HT-2 toxin |  T-2 triol |  Verrucarol |
|  |  Neosolaniol |  T-2 tetraol |  |
|  |  Diacetoxyscirpenol |  Scirpentriol |  |
|  |  |  4-acetylNIV |  |
|  |  |  DON |  |
